# Effective *MSTN* Gene Knockout by AdV-Delivered CRISPR/Cas9 in Postnatal Chick Leg Muscle

**DOI:** 10.3390/ijms21072584

**Published:** 2020-04-08

**Authors:** Ke Xu, Cheng Xiao Han, Hao Zhou, Jin Mei Ding, Zhong Xu, Ling Yu Yang, Chuan He, Fisayo Akinyemi, Yu Ming Zheng, Chao Qin, Huai Xi Luo, He Meng

**Affiliations:** Department of Animal Science, School of Agriculture and Biology, Shanghai Jiaotong University, Shanghai Key Laboratory of Veterinary Biotechnology, Shanghai 200240, China; keristina@sjtu.edu.cn (K.X.); rex_han@sjtu.edu.cn (C.X.H.); zhouhao1992@sjtu.edu.cn (H.Z.); dingjinmei@sjtu.edu.cn (J.M.D.); xz8907@sjtu.edu.cn (Z.X.); tyangly@sjtu.edu.cn (L.Y.Y.); Mars-he@sjtu.edu.cn (C.H.); fisayo@sjtu.edu.cn (F.A.); zhengyuming@sjtu.edu.cn (Y.M.Z.); qc515150910075wymq@sjtu.edu.cn (C.Q.); huangjingxiang@sjtu.edu.cn (H.X.L.)

**Keywords:** *myostatin*, CRISPR/Cas9, knockout, postnatal gene editing, transcriptome sequencing, skeletal muscle tissue, differentially expressed gene, chick

## Abstract

Muscle growth and development are important aspects of chicken meat production, but the underlying regulatory mechanisms remain unclear and need further exploration. CRISPR has been used for gene editing to study gene function in mice, but less has been done in chick muscles. To verify whether postnatal gene editing could be achieved in chick muscles and determine the transcriptomic changes, we knocked out *Myostatin* (*MSTN*), a potential inhibitor of muscle growth and development, in chicks and performed transcriptome analysis on knock-out (KO) muscles and wild-type (WT) muscles at two post-natal days: 3d (3-day-old) and 14d (14-day-old). Large fragment deletions of *MSTN* (>5 kb) were achieved in all KO muscles, and the *MSTN* gene expression was significantly downregulated at 14d. The transcriptomic results indicated the presence of 1339 differentially expressed genes (DEGs) between the 3d KO and 3d WT muscles, as well as 597 DEGs between 14d KO and 14d WT muscles. Many DEGs were found to be related to cell differentiation and proliferation, muscle growth and energy metabolism. This method provides a potential means of postnatal gene editing in chicks, and the results presented here could provide a basis for further investigation of the mechanisms involved in muscle growth and development.

## 1. Introduction

Skeletal muscle growth and development is closely related to poultry yield [1]; therefore, understanding the regulatory mechanism of chicken muscle growth and development could optimize meat production efficiency [2]. The regulatory mechanism of chicken muscle is now undergoing extensive research, as indicated by studies on the transcriptome analysis of fast-growth and slow-growth groups of the Jinghai Yellow chicken [1], chicken leg muscles at three different developmental stages [3], and chicken breast muscle at days 6 and 21 post-hatch [4]. These studies have identified some key genes (e.g., *MSTN*, *IGF1*) involved in the regulation of muscle growth and development. These findings have enhanced our understanding of the growth and development of chicken muscle, but the underlying regulatory mechanisms remain unclear and need further exploration.

One possible regulator may be the *Myostatin* (*MSTN*) gene. *MSTN*, also known as growth/differentiation factor-8 (*GDF-8*), is a member of the TGF-β superfamily that is expressed almost exclusively in skeletal muscle tissue [5], where it plays a pivotal role as a negative regulator of muscle growth and development [6,7]. In 1997, *MSTN* was first discovered and confirmed as a muscle growth inhibitor in mice [5], but it was later confirmed as highly conserved in many species [8]. The mutations and inhibition of *MSTN* expression can increase muscle mass or result in a double-muscling phenotype in many animals, including cattle [9], pigs [10], chicken [11], mice [12], and dogs [13], as well as humans [14].

The effects of *MSTN* are now being investigated using gene editing technology, an important set of techniques for studying gene function. Several researchers have generated *MSTN* knockout (KO) goats and have explored the transcriptome profile of these goats to study the effects of *MSTN* KO on muscles [15,16]. By contrast, in chickens, previous studies have focused on the association between *MSTN* gene mutations and growth traits [17,18], but few studies have investigated the molecular mechanisms of muscle growth and development using gene editing of *MSTN*. For this reason, the use of clustered regularly interspaced short palindromic repeats (CRISPR)-based genomic editing technologies now holds exceptional promise for advancing *MSTN* studies in chickens.

CRISPR technologies have greatly influenced biological, biomedical, and agricultural investigations. This technology can efficiently create numerous genetic mutations, including gene KO and knock-in mutants, to generate genetically modified cells, perform postnatal gene editing, and generate germline transgenic animals [19,20,21]. At present, this technology is not widely employed in agricultural animals like chickens to improve traits and breeding. One limitation is that the avian embryo undergoes external development and the formed egg contains 60,000 cells in the embryo, thereby making it harder to produce germline transgenic chickens. Consequently, previous applications of CRISPR-Cas9 in chickens have mainly been in vitro studies [22,23].

One viable approach in chickens is postnatal gene editing, which can also allow exploration of gene function and gene therapy [24]. For example, postnatal genome editing of mdx mice (a model of Duchenne muscular dystrophy) can partially restore dystrophin expression and improve muscle function [19,25,26]. Postnatal muscle growth is an important aspect of animal production [2]; chicks are at an important stage of growth and development. Therefore, in the present study, we knocked out the *MSTN* gene in the tibialis anterior muscle of newborn chicks using CRISPR/Cas9 technology. We then performed transcriptome sequencing of the KO and wild-type (WT) muscles. Our study aims were to confirm that CRISPR technology can be effectively used for gene editing in living chicken muscle tissues and determine the transcriptomic changes.

## 2. Results

### 2.1. MSTN Knockout (KO) in the Muscles of Chicks

**Figure 1:**
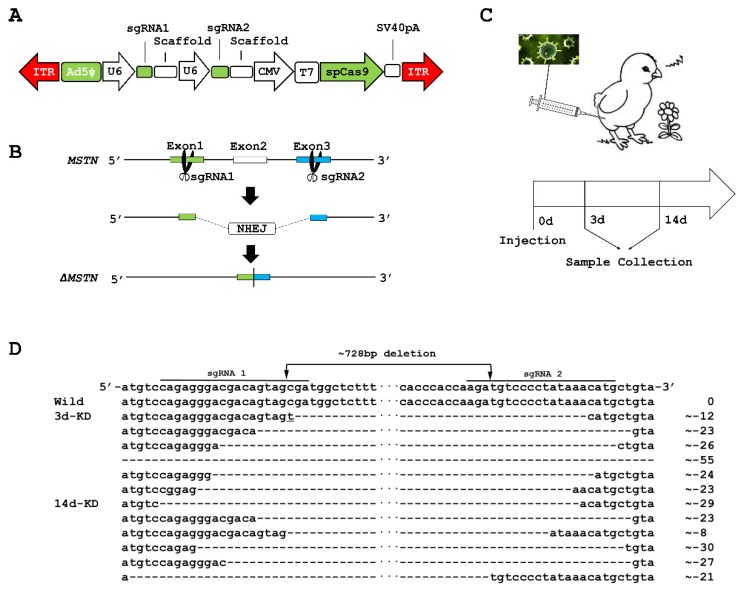
Preparation and validation of *MSTN* knockout (KO) muscles. (**A**) Schematic of the AdV-CRISPR system used in this study. (**B**) Strategy for large fragment deletion of the chick locus by non-homologous end joining (NHEJ). (**C**) Mode of AdV-CRISPR system delivery and experimental design. (**D**) Sequencing of the RT-PCR product from muscles injected with the AdV-CRISPR system.

Bioinformatics analysis showed that the *MSTN* gene is located on chromosome 7 and contains 5493 bp and three exons. We first designed single guide RNA (sgRNA) sequences targeting exon 1 and exon 3 of *MSTN*, designated as sgRNA1: CAGAGGGACGACAGTAGCGA, and sgRNA2: CATGTTTATAGGGGACATCT. We then used an adenovirus (AdV) vector to co-express SpCas9 and the pair of sgRNAs (Figure 1A). Figure 1B shows the strategy for using CRISPR/Cas9-mediated non-homologous end joining (NHEJ) to create 5093 bp deletions in the chick genome, which means 728 bp deletions of cDNA. We validated the *MSTN* gene-editing function of the AdV-CRISPR system by transducing it into DF-1 cells and performing PCR using DNA extracted from DF-1 cells. Agarose gel electrophoresis revealed a 550 bp band for the *MSTN* product in the cells transduced with the AdV-CRISPR system after a predicted deletion of 5093 bp, whereas the control cells had no band (Figure S1). The PCR products were cloned into the TA cloning vector, and the sequencing data from individual clones confirmed the large fragment deletion (Figure S2). These results validated the gene-editing function of the AdV-CRISPR system for *MSTN* in chicken cells.

We delivered the AdV-CRISPR system into the tibialis anterior muscle of chicks and then collected the muscle tissues from the chicks at the 3-day-old (3d) and 14-day-old (14d) stages (Figure 1C). We performed reverse transcription PCR (RT-PCR), employing RNA isolated from the muscles. The RT-PCR products were also cloned into the TA cloning vector, and the sequencing data from individual clones confirmed that all injected AdV-CRISPR system muscles were identified as having the deletion mutant, with the number of deleted nucleotides in the mRNA ranging from 736 to 783 nucleotides (Figure 1D). Determination of the expression level of *MSTN* using real-time PCR revealed a significant downregulation of *MSTN* expression (*p*-value < 0.001) in the 14d KO muscles compared to the 14d wild-type (WT) muscles (Figure S3). Deletion of 736 to 783 nucleotides in mRNA can induce deletion of up to 261 amino acids in the *MSTN* protein.

### 2.2. Differentially Expressed Genes (DEGs) in KO Muscles

In this study, transcriptome sequencing was used to detect the mRNA expression profiles of KO muscles and WT muscles. A total of twelve RNA libraries were constructed from the total RNA isolated from the following four tibialis anterior muscle sample groups: the 3d KO group, 3d WT group, 14d KO group, and 14d WT group (three replicates in each group). In total, approximately 529 million clean reads were obtained and about 92% of the sequence reads per sample could be mapped to the chicken reference genome (galgal6), suggesting a good sequence quality.

**Figure 2:**
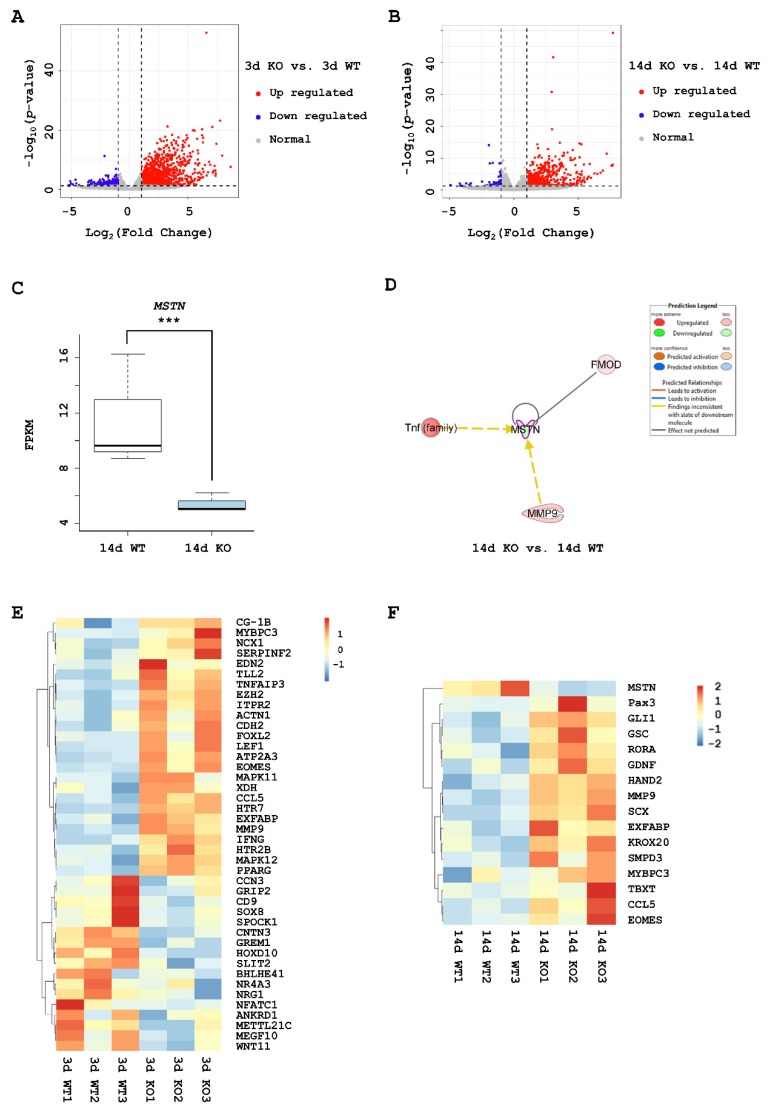
Differentially expressed genes (DEGs) from RNA-Seq data. (**A**) Volcano plot reveals significant differentially expressed genes in the 3d KO vs. 3d wild-type (WT) groups. (**B**) Volcano plot of significant differentially expressed genes of the 14d KO vs. 14d WT groups. *** *p*-value < 0.001. (**C**) The expression of *MSTN* in the 14d KO and 14d WT groups. (**D**) DEGs associated with *MSTN* in the 14d KO vs. 14d WT groups. (**E**) Heatmap hierarchical clustering revealed the DEGs related to skeletal muscle tissue development in 3d KO vs. 3d WT groups. (**F**) Heatmap hierarchical clustering revealed the DEGs related to skeletal muscle tissue development in the 14d KO vs. 14d WT. This result also has some limitations, because the KO groups were injected with AdV-CRISPR, while the WT groups were injected with phosphate buffered saline (PBS).

Principal components analysis (PCA) showed clusters of similar samples (Figure S4). The differences in the transcriptome of *MSTN* KO muscle were analyzed by comparing 3d KO vs. 3d WT and 14d KO vs. 14d WT. The comparison of 3d KO and 3d WT revealed 1339 significantly differentially expressed genes, including 1110 upregulated and 229 downregulated genes in the 3d KO samples (Figure 2A, Table S1). Differential gene expression analysis between 14d KO and 14d WT chicks showed 597 significantly differentially expressed genes, including 545 upregulated genes and 52 downregulated genes (Figure 2B, Table S2).

The expression of *MSTN* was downregulated in the KO groups compared to the WT groups at 3d and 14d, with the difference at 14d being significant (*p*-value < 0.001) (Figure 2C). Ingenuity Pathway Analysis (IPA; Qiagen, California, the US) revealed 14 factors interacting with *MSTN* in 3d KO vs. 3d WT (Figure S5), and 3 factors interacting in 14d KO vs. 14d WT (Figure 2D). *FMOD*, *MMP9*, and *Tnf* (*family*) were common molecules interacting with *MSTN*. IPA showed that *MSTN* was related to atrophy of the tibialis anterior and the myotube. *MSTN* and *MMP9* participate in myofibroblast differentiation, myofiber regeneration, muscle fibrosis, and alterations in the mass of the tibialis anterior. *MSTN* and *FMOD* impact the formation and strength of muscle. *MSTN* and the *Tnf* (*family*) genes have roles in the proliferation of fibroblasts and muscle cells.

Overall, numerous DEGs were related to skeletal muscle tissue development: 42 in the 3d KO vs. 3d WT group and 16 in the 14d KO vs. 14d WT group. Of these, five DEGs, including *MMP9*, *EOMES* and *Ex-FABP*, were observed in both comparisons. Hierarchical clustering showed the expression profiles of these genes related to skeletal muscle tissue development (Figure 2E,F).

### 2.3. Gene Ontology (GO) and Pathway Analyses of DEGs

**Figure 3:**
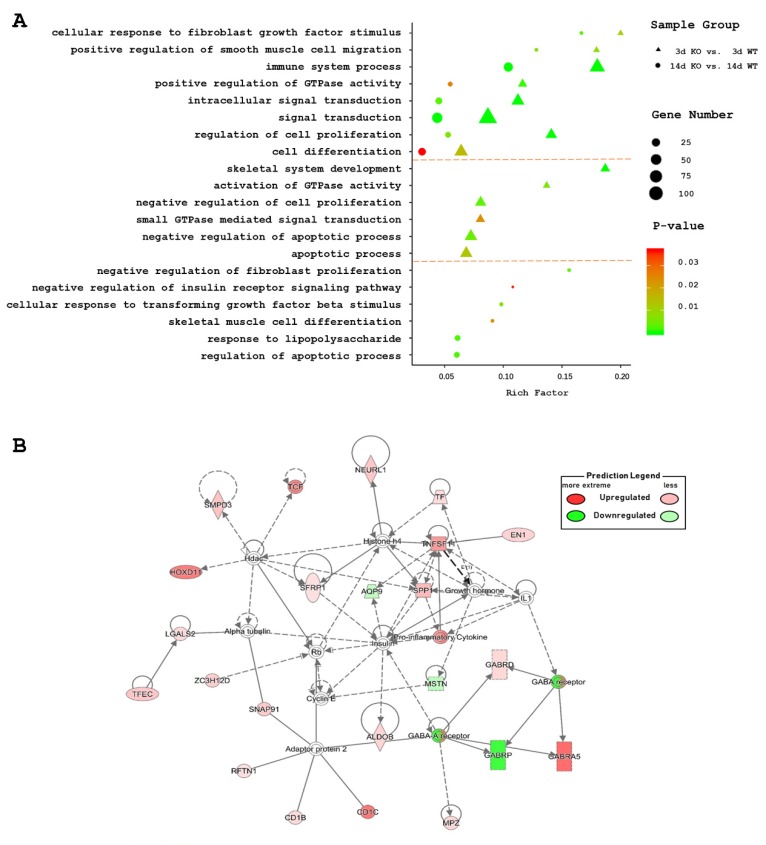
Representative enriched gene ontology functional classifications and associated network of differential expression genes (DEGs) between *MSTN* knockout (KO) and wild-type (WT) muscles. (**A**) Representative gene ontology (GO) enrichment terms of DEGs in the 3d KO vs. 3d WT groups and 14d KO vs. 14d WT groups. (**B**) Gene network containing DEGs related to Neurological Disease, Organismal Injury and Abnormalities, and Skeletal and Muscular Disorders. This result also has some limitations, because the KO groups were injected with AdV-CRISPR, while the WT groups were injected with PBS.

The Database for Annotation, Visualization and Integrated Discovery (DAVID) website was used to identify the DEG functions. Overall, 335 significantly enriched entries were identified in the biological process category in the 3d KO vs. 3d WT groups, and 167 in the 14d KO vs. 14d WT groups (Tables S3 and S4). Most biological process-enriched items in these two comparisons were significantly associated with cell differentiation and proliferation, muscle growth and development, energy metabolism, apoptosis process, immune system, and signal transduction (Figure 3A). In 3d KO vs. 3d WT, skeletal system development was also enriched. The negative regulation of the insulin receptor signaling pathway was enriched in the 14d KO vs. 14d WT. Comparison of the gene lists of 14d KO and 14d WT indicated an involvement of *MSTN* in several GO terms, including negative regulation of the insulin receptor signaling pathway, regulation of the apoptotic process, and cytokine activity (GO:0045893, GO:0042981, GO:0045471, GO:0046627).

The pathways associated with the DEGs were identified by subjecting the DEGs to Ingenuity Pathway Analysis (IPA) analyses. The most relevant molecular and cellular functions in skeletal muscles comparisons between KO and WT at 3d and 14d included cellular development, cellular growth and proliferation, cellular function and maintenance, cell-to-cell signaling and interaction, and cellular movement. IPA analysis also generated 32 and 15 canonical pathways, about cellular growth, proliferation, and development, in the 3d KO vs. 3d WT and the 14d KO vs. 14d WT groups respectively (Tables S5 and S6). The 15 pathways enriched in the 14d KO vs. 14d WT groups were also enriched in 3d KO vs. 3d WT. In both comparisons, the most relevant of the 15 pathways identified were the two related to muscle growth: the PDGF signaling pathway and the STAT3 signaling pathway. The PDGF signaling pathway is related to rapid growth and greater muscle mass [27,28], whereas the STAT3 signaling pathway is a critical mediator of myoblast proliferation [29]. The IPA analysis identified a significant gene network for DEGs in the 14d KO vs. 14d WT and implicated the *MSTN* gene. This network was associated with Skeletal and Muscular Disorders, as well as with Neurological Disease and Organismal Injury and Abnormalities (Figure 3B).

## 3. Discussion

The chicken has been used as a model organism for many years, and it also provides the human diet across the globe with large amounts of protein [30]. Genetically modified chicken is now important in many areas, including the improvement of production of meat and eggs, enhancement of disease resistance, and establishment of models for studying the functions of specific genes. Postnatal gene editing has been used to study gene function and realize gene therapy in postnatal mice [24,31]. In this study, we show that programmable CRISPR complexes can be delivered to skeletal muscle in chicks, where they mediate targeted gene modification and reduce *MSTN* mRNA expression. Although in vivo CRISPR-mediated *MSTN* gene editing has been applied in mammals such as goats [15], pigs [32], sheep [33], and rabbits [34], no reports have yet emerged on the use of *MSTN*-CRISPR in postnatal chicks.

Muscle growth and development are regulated by genes, biological processes and signal pathways [35,36]; therefore, the underlying regulatory mechanisms are a hot topic of interest. In the present research, transcriptome sequencing was used to analyze the differentially expressed genes between *MSTN* knock-out (KO) muscles and wild-type (WT) muscles. A total of approximately 529 million clean reads were obtained, and the expression of *MSTN* was downregulated at 3d and 14d. Three DEGs (*COL1A1*, *COL1A2*, and *COL3A1*) in the 3d KO vs. 3d WT groups have been reported in previous studies [37,38]. Significant changes occurred in the expressions of *MYH15* in 3d KO vs. 3d WT, and *SCD* in 14d KO vs. 14d WT, in agreement with results reported previously for *MSTN*-KO goats [16]. Our study identified several DEGs that interacted with *MSTN*. One was *FMOD*, which is not only a new regulator of *MSTN* during myoblast differentiation but can also circumvent the inhibitory effect of *MSTN* and trigger myoblast differentiation [39]. Another was *MMP9*, which plays a role in regulating *MSTN* and causes muscle hypertrophy when genetically manipulated [40,41]. In the present research, through GO enrichment analysis, many genes, such as, *SCX, Pax3, VTN, EOMES, PAX5, MYC, NOX4, P2RY6, ITGA2, CCL5, PGR, SRC, P2RY6, TNFRSF25, TCF7, CXCL13, SFRP1* etc., were found to be involved in the skeletal muscle cell differentiation, positive regulation of smooth muscle cell migration, regulation of cell proliferation, and negative regulation of cell growth. *SCX* can mediate bone ridge patterning during musculoskeletal assembly [42], and *Pax3* plays a key role during postnatal myogenesis [43]. *VTN* encodes proteins of the extracellular matrix (ECM), and the ECM forms the structural basis of muscle cells [44]. *SFRP1* prevents myoblast differentiation [45]. In addition, the IPA analysis underscored the importance of two main canonical pathways involving muscle growth—the STAT3 signaling pathway and PDGF signaling pathway—in the current study.

The study findings showed upregulation of almost all genes related to positive regulation of GTPase activity, activation of GTPase activity, and small GTPase mediated signal transduction in the KO group compared with the WT group. *PRCP* in 3d KO vs. 3d WT and *IRF4* in 14d KO vs. 14d WT are important regulators of energy metabolism [46,47]. Meanwhile, *MSTN* appears to function as a potential regulator of energy metabolism in mice [48], and previous studies on the use of *MSTN* protein to treat chicken fetal myoblasts have also shown an altered expression of genes involved in energy metabolism [49].

This approach also has some limitations, because the KO groups were injected with AdV-CRISPR, while the WT groups were injected with phosphate buffered saline (PBS). A better control would be injection of AdV loaded with SpCas9, as the transcriptomic changes could be directly attributed to the *MSTN* KO. In future studies, we will use a more suitable control to provide more definitive insights.

In the present study, we used gene editing to create *MSTN* KO muscles, and we analyzed the DEGs between KO muscles and WT muscles at 3d and 14d. Large fragment deletions of *MSTN* were achieved in all KO muscles, and the expression of *MSTN* was downregulated at 3d and 14d. GO enrichment and IPA analyses revealed many genes that interacted with *MSTN*, or that were involved in cell differentiation and proliferation, muscle growth and development, and energy metabolism. The demonstration of successful CRISPR/Cas9 gene editing in newborn chicks opens up new avenues for these types of studies in avian species. The results of this study will provide a basis for further investigations into the mechanisms involved in muscle growth and development.

## 4. Materials and Methods

### 4.1. Plasmid Construction

In order to achieve large fragment deletion, a pair of sgRNAs directed at exon 1 and exon 3 of *MSTN* were designed by CRISPR DESIGN (http://crispr.mit.edu/). An adenoviral (AdV) vector expressing SpCas9, sgRNA1 and sgRNA2 was produced and purified by the ViGene Biosciences (Shandong, China), and designated the pAdM-U6-gRNA-CMV-spCas9 vector. Titers were expressed as plaque forming units per mL (pfu/mL) and the AdV titers used for injection in chickens were 1.04 × 10^11^ pfu/mL.

### 4.2. Feasibility Assessment of the AdV-CRISPR System in DF-1 Cells

DF-1 cells were cultivated in Dulbecco’s Modified Eagle Medium (DMEM), supplemented with 10% fetal bovine serum at 37 °C, 5% CO_2_. The pAdM-U6-gRNA-CMV-spCas9 vector was gently dropped into a six-well plate containing chicken DF-1 cells at 80% confluency. The cells were transduced at a multiplicity of infection (MOI) of 1000. After incubation for 6 h, the cells were gently washed twice with phosphate buffered saline (PBS), and fresh culture medium was added.

One day after transduction, genomic DNA was extracted for PCR using the following primers: Primer 1F: TAGAACTGAAAGAAAAG; and Primer 3R: TTCAAAGATGGATGAGGGGATA. The PCR conditions were: 94 °C for 5 min; 35× (94 °C for 30 s, 60 °C for 30 s, 72 °C for 45 s); 72 °C for 10 min; followed by 4 °C for 30 min. Large fragment deletion was confirmed by cloning PCR amplicons into the pMD19-T vector (TaKaRa, Dalian, China) and sequencing.

### 4.3. Fertilized Eggs, Incubation, and Chick Breeding

Specific pathogen-free fertilized eggs were obtained from Sais Poultry Co. Ltd., Jinan, China. The eggs were incubated in an Ova-Easy Advance Series II Digital Cabinet Egg Incubator (Brinsea, UK) within a sterilized room at 37.8 °C and 55–65% humidity. All chickens were bred in the Animal Husbandry and Veterinary Research Institute (Shanghai Academy of Agricultural Science, Shanghai, China) under standardized conditions.

### 4.4. Intramuscular Injection of the AdV-CRISPR System

Skeletal muscle was transduced in vivo by injecting the right tibialis anterior of newborn chicks with the AdV-CRISPR (1.04 × 10^9^ pfu/chicken). We injected PBS into the left tibialis anterior as a control. After 3 days and 14 days, the chicks were euthanized by diethyl ether inhalation. The tibialis anterior muscles were collected in liquid nitrogen for subsequent experiments (reverse transcription PCR, real-time PCR and RNA-Seq).

### 4.5. Detection of Mutations in the MSTN Gene

Muscle tissue blocks were dissolved in Trizol reagent (Invitrogen, Carlsbad, CA, USA) for total RNA extraction. RNA was reverse transcribed into cDNA using a PrimeScript™ RT Reagent Kit with gDNA Eraser (TaKaRa, Dalian, China), according to the manufacturer’s instructions.

We then amplified the target genomic region by PCR. The PCR assays contained 25 μL Taq PCR Master Mix (Sangon Biotech, Shanghai, China), 2 μL 10μM primer (RT1F: TGCTTGTACGTGGAGACAGAATAC, RT3R: ACCATGGCTGGTATCTTTCCATA), and ddH2O to 50 μL. PCR conditions were: 94 °C for 4 min; 35 × (94 °C for 30 s, 60 °C for 30 s, 72 °C for 45 s); 72 °C for 10 min; followed by 4 °C for 30 min. The RT-PCR products of *MSTN* knockout (KO) muscle were cloned and sequenced to confirm the elimination of *MSTN*.

We also performed real-time PCR and chose the β-actin gene as reference house-keeping gene. SYBRR Premix Ex Taq^TM^ (Tli RNaseH Plus) (TaKaRa, Dalian, China) was used to perform the qPCR reactions in a StepOne Real-Time PCR System, with a 20 μL reaction system comprising 10 μL of SYBRR Green Premix Ex Taq II (2×), 0.8 μL of each of the forward and reverse primers (10 μM), 0.4 μL of ROX Reference Dye (50×), 2 μL of cDNA and 6 μL of distilled water. The primers for *MSTN* were the same as those used for RT-PCR. The primers for β-actin were 2F: GAGAAATTGTGCGTGACATCA, and 2R: CCTGAACCTCTCATTGCCA. The program was 95 °C for 30 s; followed by 40 cycles of 95 °C for 5 s, 60 °C for 30 s; and ended with a melting curve analysis. The 2^−ΔΔ*C*t^ method was used to calculate the relative expression levels between the KO and WT groups.

### 4.6. Transcriptome Sequencing and Differentially Expressed Gene (DEG) Analysis

RNA was extracted from the tibialis anterior muscles and sent to Shanghai Personal Biotechnology Corporation (Shanghai, China) for mRNA purification, library preparation and sequencing. A total of 12 samples (three replicates for each group) were sequenced on the Illumina Hiseq 2500 platform. After filtering the raw data and removing low-quality reads, the selected clean reads were mapped to the Gallus gallus genome (GRCg6a) using HISAT2(http://ccb.jhu.edu/software/hisat2/index.shtml) and the transcript expression was calculated using HTSeq v0.6.1.

We used the DESeq2 R package to analyze the differentially expressed genes (DEGs). Genes with *p*-value < 0.05 and |log_2_ fold change| > 1 were considered to be differentially expressed.

### 4.7. Gene Ontology (GO) and Pathway Analysis of Differentially Expressed Genes (DEGs)

GO enrichment analysis of the DEGs was performed using the DAVID 6.8 Functional Annotation Tool (https://david.ncifcrf.gov/). GO terms with *p*-value < 0.05 were considered significantly enriched by DEGs.

We uploaded DEGs onto the IPA package and analyzed the data using default settings for canonical pathway analysis, network discovery and determination of molecules interacting with *MSTN* by overlay. *p*-value < 0.05 was considered significantly different.

### 4.8. Ethics Statement

All animal experiments were approved by the Animal Ethics Committee at Shanghai Jiao Tong University in China, approval No. 202002005 (10 February 2020).

## Supplementary Materials

Supplementary Materials can be found at https://www.mdpi.com/1422-0067/21/7/2584/s1.

## Abbreviations

*MSTN*	*Myostatin*
AdV	adenovirus
KO	knockout
WT	wild-type
3d	3-day-old
14d	14-day-old
DEGs	differentially expressed genes
CRISPR	clustered regularly interspaced short palindromic repeats
sgRNA	single guide RNA
NHEJ	non-homologous end joining
RT-PCR	reverse transcription polymerase chain reaction
PCA	Principal components analysis
IPA	Ingenuity Pathway Analysis
GO	Gene ontology
DAVID	Database for Annotation, Visualization and Integrated Discovery
