# Supplementary material for: Effective MSTN Gene Knockout by AdV-Delivered CRISPR/Cas9 in Postnatal Chick Leg Muscle
Source: Int J Mol Sci. 2020 Apr 8;21(7):2584. doi: 10.3390/ijms21072584 (PMC7177447; doi:10.3390/ijms21072584)
Supplement: Supplementary file 1 [file ijms-21-02584-s001.zip › ijms-755222-proofdone sup/ijms-755222-supplementary/Supplementary/Supplementary Material.docx]

*Supplementary Material*

**Supplementary Figures**


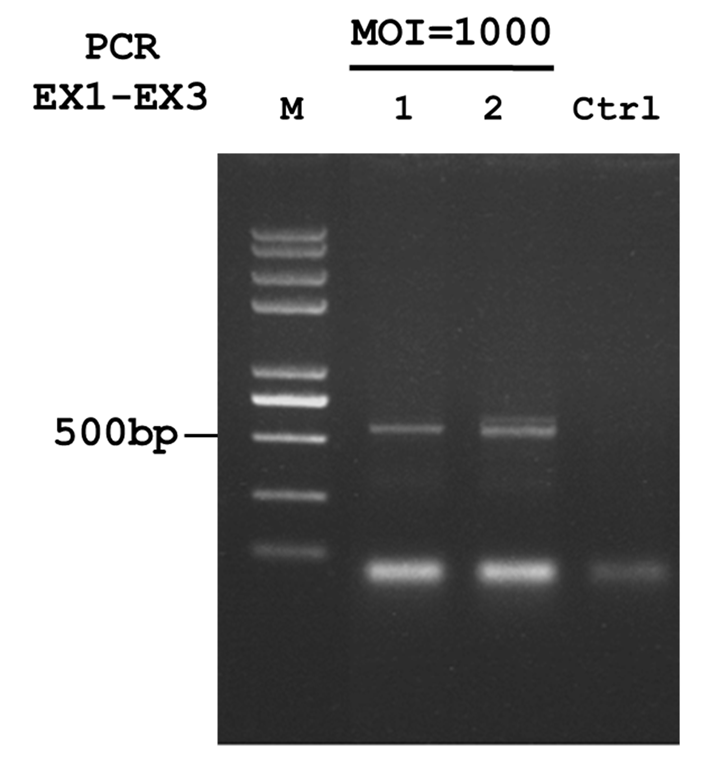


**Figure S1.** Electrophoretogram of the results of cell validation by PCR.


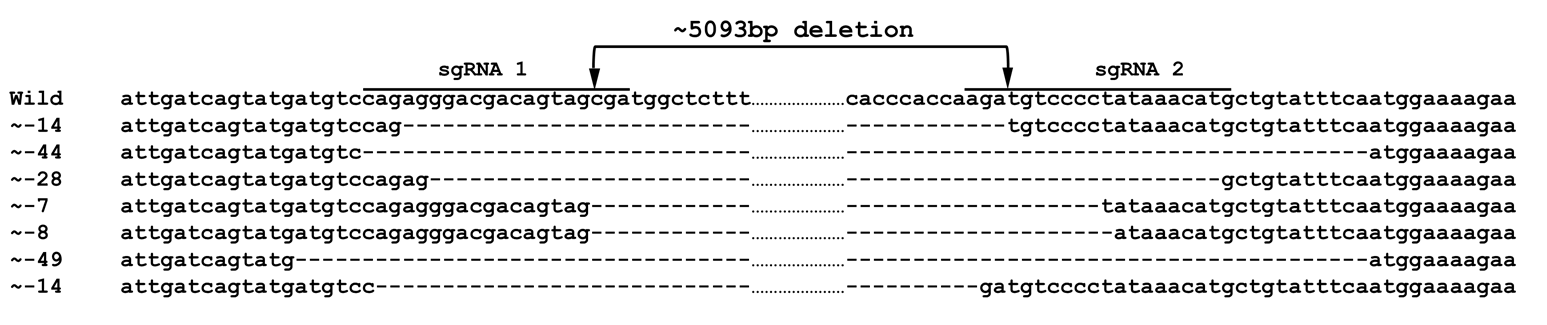


**Figure S2.** Sequencing of the PCR product from cells transduced with the AdV-CRISPR system.


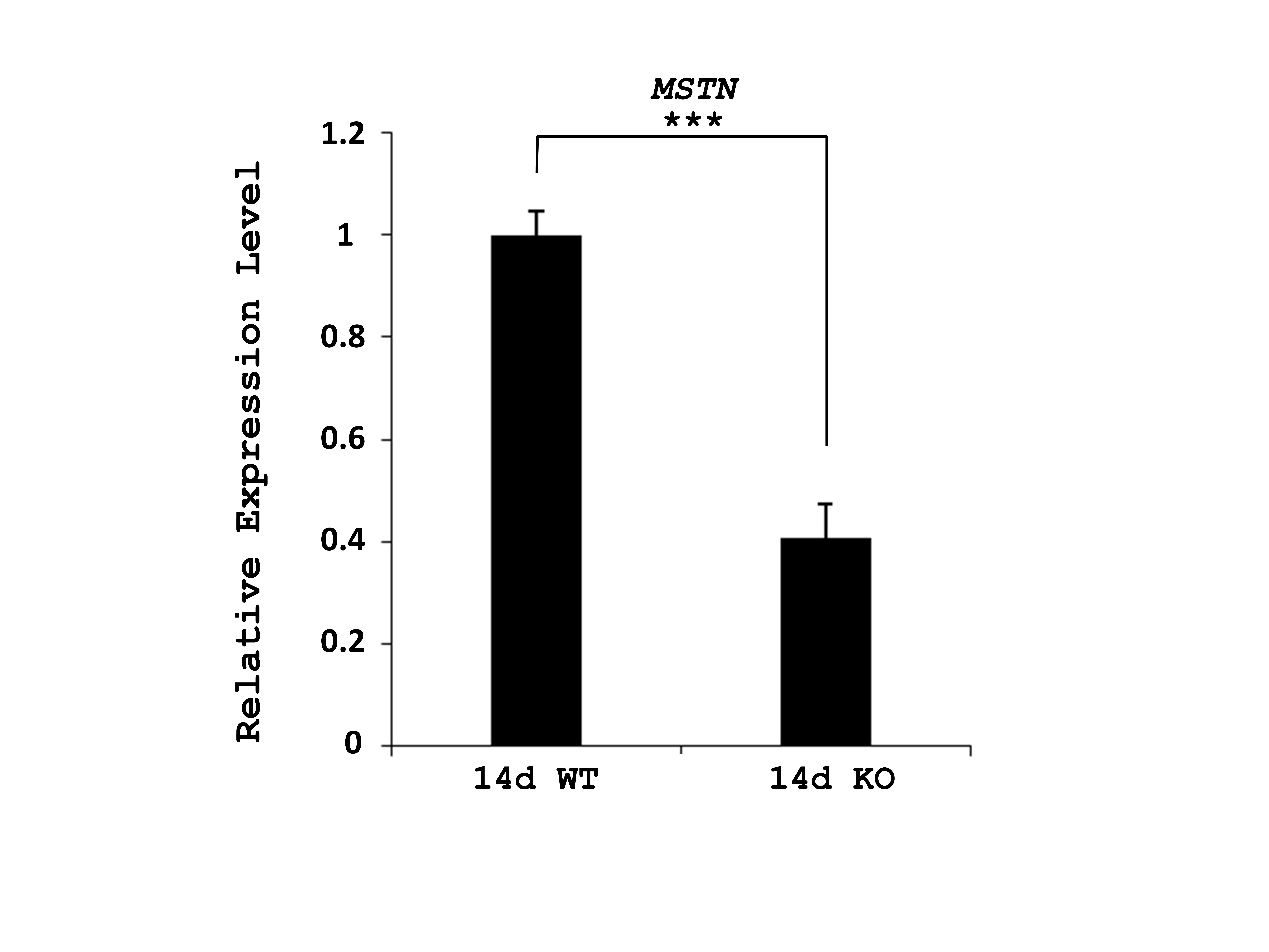


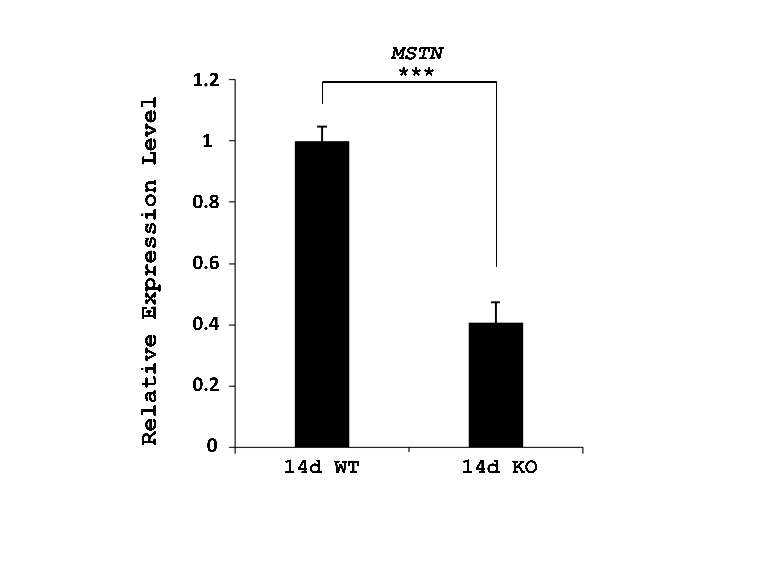


**Figure S3.** Expression level of *MSTN* in 14d WT and 14d KO groups detected by real-time PCR.


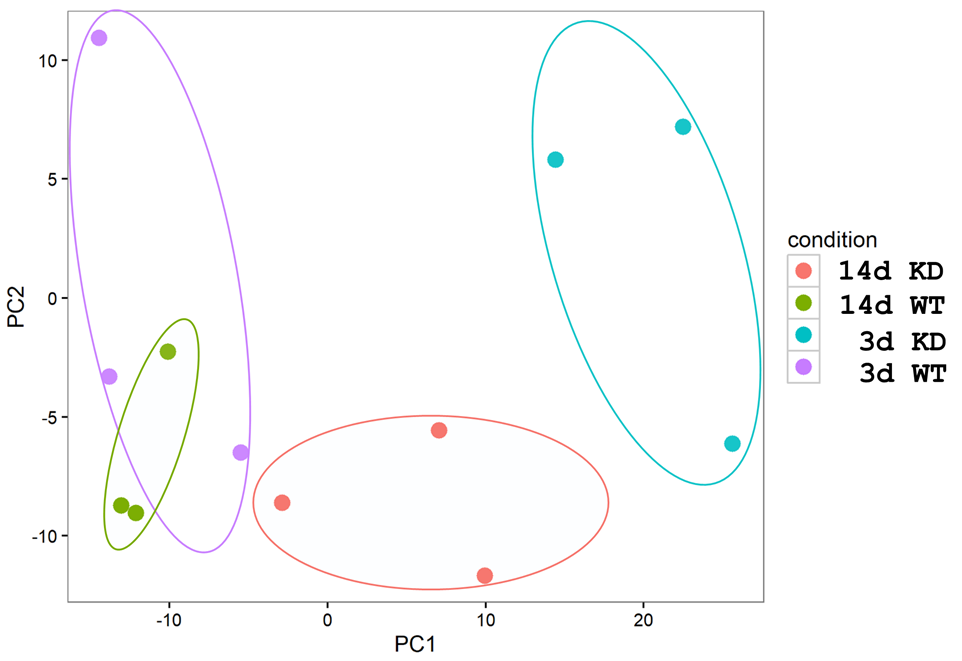


**Figure S4.** Principal component analysis for all chick muscles at both time points. Principal component 1 (PC1) and Principal component 2 (PC2) were identified by logarithm transformation in DESeq2 at two time points.


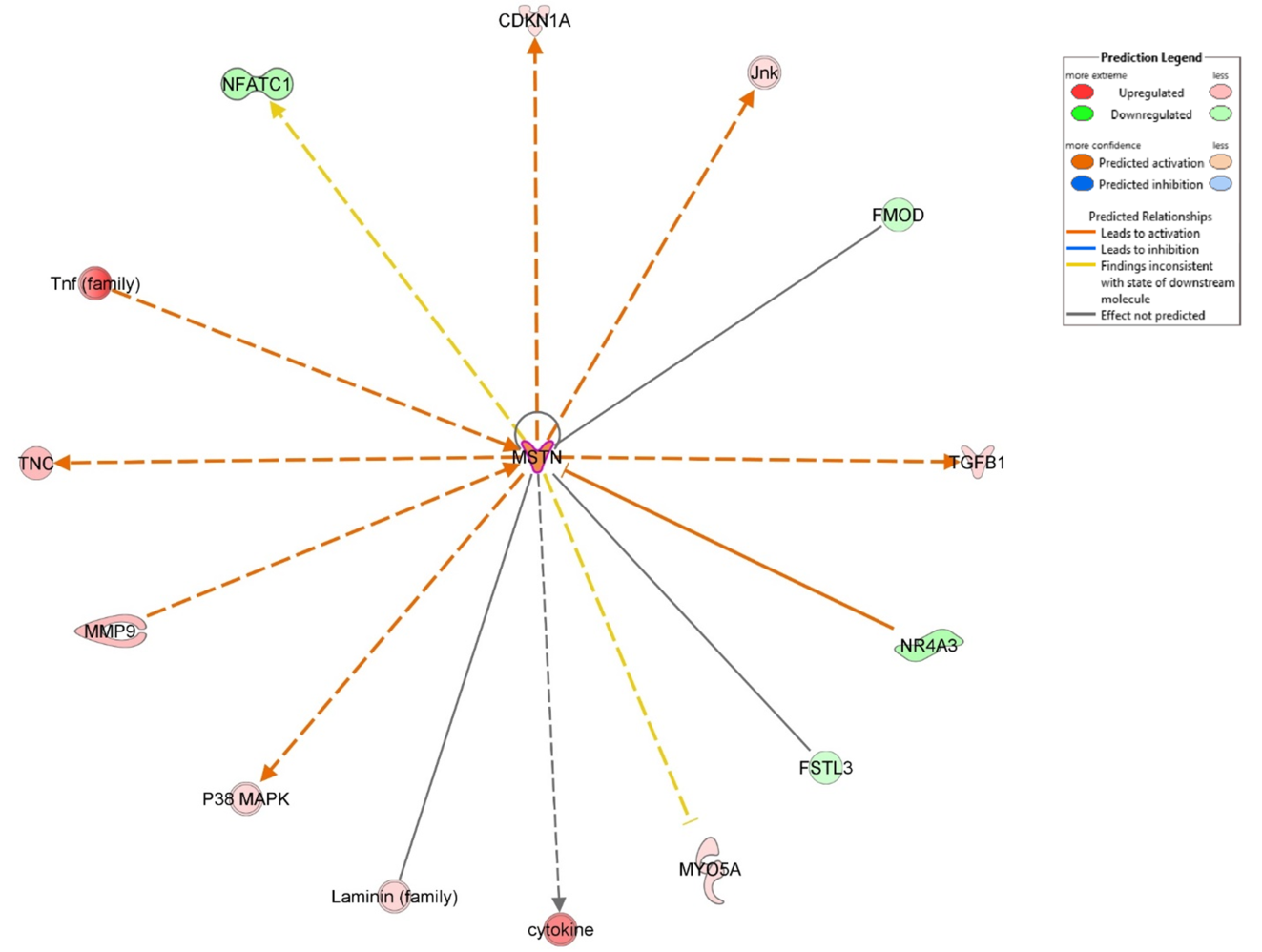


**Figure S5.** Differentially expressed genes associated with *MSTN* in the 3d KO vs. 3d WT groups.
